# Network Characteristics and Visualization of COVID-19 Outbreak in a Large Detention Facility in the United States — Cook County, Illinois, 2020

**DOI:** 10.15585/mmwr.mm6944a3

**Published:** 2020-11-06

**Authors:** Uzay Kırbıyık, Alison M. Binder, Isaac Ghinai, Chad Zawitz, Rebecca Levin, Usha Samala, Michelle Bryant Smith, Jane Gubser, Bridgette Jones, Kate Varela, Josh Rafinski, Anne Fitzgerald, Peter Orris, Alex Bahls, Sharon Welbel, Connie Mennella, Stephanie R. Black, Paige A. Armstrong

**Affiliations:** ^1^Division of Laboratory Systems, Center for Surveillance, Epidemiology, and Laboratory Services, CDC; ^2^Division of Vector-Borne Diseases, National Center for Emerging and Zoonotic Infectious Diseases, CDC; ^3^Epidemic Intelligence Service, CDC; ^4^Chicago Department of Public Health, Chicago, Illinois; ^5^Cermak Health Services, Chicago, Illinois; ^6^Cook County Health, Oak Forest, Illinois; ^7^Cook County Sheriff’s Office, Chicago, Illinois; ^8^University of Illinois at Chicago.

Correctional and detention facilities have been disproportionately affected by coronavirus disease 2019 (COVID-19) because of shared space and movement of staff members and detained persons within facilities (*1*,*2*). During March 1–April 30, 2020, at Cook County Jail in Chicago, Illinois, >900 COVID-19 cases were diagnosed across all 10 housing divisions, representing 13 unique buildings.^†^ Movement within the jail was examined through network analyses and visualization, a field that examines elements within a network and the connections between them. This methodology has been used to supplement contact tracing investigations for tuberculosis and to understand how social networks contribute to transmission of sexually transmitted infections (*3*–*5*). Movements and connections of 5,884 persons (3,843 [65%] detained persons and 2,041 [35%] staff members) at the jail during March 1–April 30 were analyzed. A total of 472 (12.3%) COVID-19 cases were identified among detained persons and 198 (9.7%) among staff members. Among 103,701 shared-shift connections among staff members, 1.4% occurred between persons with COVID-19, a percentage that is significantly higher than the expected 0.9% by random occurrence alone (p<0.001), suggesting that additional transmission occurred within this group. The observed connections among detained persons with COVID-19 were significantly lower than expected (1.0% versus 1.1%, p<0.001) when considering only the housing units in which initial transmission occurred, suggesting that the systematic isolation of persons with COVID-19 is effective at limiting transmission. A network-informed approach can identify likely points of high transmission, allowing for interventions to reduce transmission targeted at these groups or locations, such as by reducing convening of staff members, closing breakrooms, and cessation of contact sports.

All detained persons with data available for at least one bed assignment at Cook County Jail during March 1–April 30, 2020, were identified using records provided by Cook County Sheriff’s Office (CCSO), and Cermak Health Services. CCSO staff members who worked at least one shift at the jail during the same period were also included. A case of COVID-19 was defined as detection of SARS-CoV-2 by real-time reverse transcription–polymerase chain reaction (RT-PCR) in a specimen from a detained person, and, among staff members, as reported COVID-19–compatible symptoms or detection of SARS-CoV-2 in specimens by real-time RT-PCR. Detained persons who reported symptoms or who were a close contact of someone with a positive test result were tested; those who were not tested (2,763; 72%) or who received a negative test result (608; 16%) were grouped together for analyses and visualizations. Although staff members were not systematically tested, they were required to report symptoms of COVID-19 or receipt of positive test results immediately to CCSO; staff members reporting positive test results (confirmed case) or symptoms (probable case) were considered to have COVID-19. Staff member test results were confirmed through the Illinois National Notifiable Disease Surveillance System.

## Description of Networks

In the person-division networks, divisions represent housing buildings within the jail. Connections occur between persons (detained persons or staff members) and the divisions to which they are assigned, either for housing or a work shift. The number of connections between persons and divisions is calculated based on a value of one for each new bed assignment for detained persons or shifts within a given division for staff members.

To determine whether the number of connections to a given division was associated with a higher proportion of cases among staff members and detained persons, the linear correlation between the proportion of persons meeting the case definition during the study period and the number of connections was compared for each division, representing all persons linked to each division through at least one work shift (staff member) or cell or bed assignment (detained person). The strength of correlation was determined by calculating the correlation coefficients for each line of best fit, with statistical significance assessed at α = 0.05.

The person-person networks show persons present at the same location and time based either on shift or bed assignment. To determine whether connections between persons with COVID-19 in both groups were occurring more frequently than expected, the proportion of persons meeting the case definition was used to calculate ratios of observed-to-expected proportions of positive-positive, positive-negative (or not known to be positive), and negative-negative connections, and these ratios were compared using chi-squared tests of independence; significance was assessed at α = 0.05.

A joined network of detained persons and staff members was constructed but did not demonstrate clear patterns of clustering or spread. Detained person and staff member networks, displayed separately as unique patterns for each network, were more easily visualized. Data management and analyses were conducted using SAS (version 9.4; SAS Institute) and R (version 3.6.2; The R Foundation) statistical software; visualizations were performed using Gephi (version 0.9.2; The Gephi Consortium). This activity was reviewed by CDC and was conducted consistent with applicable federal law and CDC policy.^§^

The average daily census of detained person during March 1–April 30, 2020, was 4,884, with 3,834 (79%) included in the analysis based on availability of cell or bed assignment. Among the 1,080 detained persons tested during the same period, 472 (12.3%) received a positive SARS-CoV-2 test result. CCSO had 2,370 staff members assigned to Cook County Jail on March 1, 2020, most of whom worked on site. During the outbreak, 270 staff members were added, totaling 2,640. Among these, 2,041 (77%) were included in the analysis based on availability of shift and division assignments, 198 (9.7%) of whom had COVID-19; staff members with negative test results could not be identified through available data sources. During the outbreak, interventions were used to limit spread, including cessation of visitation (March 15), suspension of programmatic activities (March 23), conversion of cells to single occupancy, and universal masking for staff members (April 2) and detained persons (April 13).

**FIGURE 1:**
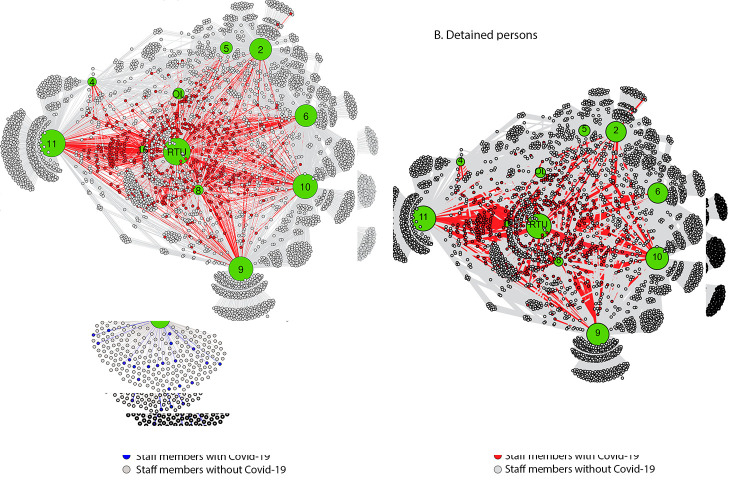
Visualization of staff members (A)* and detained persons (B)^†^ epidemiologically linked to an outbreak of COVID-19^§^ using person-division networks^¶^ — Cook County Jail, Illinois, March 1–April 30, 2020 **Abbreviations:** COVID-19 = coronavirus disease 2019; OL = offsite location; RTU = residential treatment unit. * Staff members–division network includes 1,843 persons who did not have COVID-19 (gray) and 198 with COVID-19 (blue) as reported to the Cook County Sheriff’s Office. Lines (connections) are colored according to the same color scheme. ^†^ Detained persons–division network includes 3,371 persons without COVID-19 (gray) and 472 persons with COVID-19 (red). Lines (connections) are colored according to the same color scheme. ^§^ COVID-19 cases were defined as detection of SARS-CoV-2 (the virus that causes COVID-19) by real-time reverse transcription–polymerase chain reaction (RT-PCR) testing in specimens from detained persons, and among staff members, as reported symptoms or SARS-CoV-2 positive RT-PCR test results. ^¶^ Numbers and letters in large circles within figure represent the individual housing divisions; circle sizes correlate to the number of connections (e.g., a larger circle indicates higher number of connections). Location of division node is not representative of the geographic location of the division on-site at the jail.

**Person-division networks.** Visualization of networks among detained persons and staff members indicates that COVID-19 cases occurred in all jail divisions. The staff member network did not demonstrate a discernable pattern with distribution of persons with COVID-19 throughout divisions (Figure 1). Detained persons with COVID-19 appeared to cluster at division 8/residential treatment unit (RTU) and division 16, both of which were used for medical isolation, and offsite locations (e.g., hospitalizations) (Figure 1).

**FIGURE 2:**
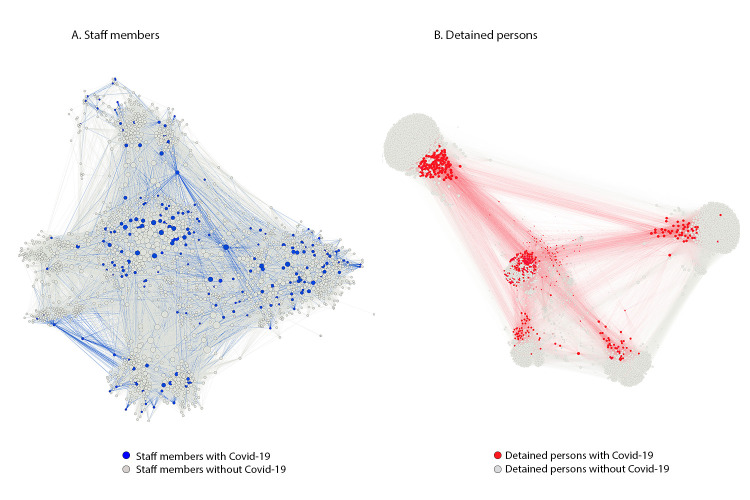
Visualization of staff members* (A) and detained persons^†^ (B) epidemiologically linked to an outbreak of COVID-19^§^ using person-person networks — Cook County Jail, Illinois, March 1–April 30, 2020 **Abbreviation:** COVID-19 = coronavirus disease 2019. *Staff members–person-person network includes 103,701 connections between 1,843 persons who did not have COVID-19 (gray) and 198 persons with COVID-19 as reported to the Cook County Sheriff’s Office (blue). Lines (connections) are colored according to the same color scheme. Observed positive-positive connections were higher than expected (n = 1,420 [1.4%] versus n = 976 [0.9%], p<0.05). Most observed connections were between persons who did not have positive test results (n = 83,813, 80.8%). ^†^ Detained persons–person-person network includes 1,214,462 connections between 3,371 persons without COVID-19 (gray) and 472 persons with COVID-19 (red). Connections are colored according to the same color scheme. Observed positive-positive connections were higher than expected (n = 39,141 [3.2%] versus n = 18,320 [1.5%], p<0.05). When excluding connections associated with persons in medical isolation or at off-site locations (e.g., residential treatment unit, division 16, and off-site locations, the rate of observed connections is significantly less than expected (n = 11,017 [1.0%] versus n = 12,165 [1.1%], p<0.05). In March, as the number of persons with COVID-19 were increasing, the mean number of interactions between staff members with COVID-19 (n = 377) was significantly higher than that of staff members without COVID-19 (n = 321) [p<0.001]. This difference was not seen in April when cases were declining. ^§^ COVID-19 cases were defined as detection of SARS-CoV-2 (the virus that causes COVID-19) by real-time reverse transcription–polymerase chain reaction (RT-PCR) testing in specimens from detained persons, and among staff members, as reported symptoms or SARS-CoV-2 positive RT-PCR test results.

**Person-person networks.** Overall, 103,701 shared shift connections occurred among staff members, 1.4% of which were between staff members with COVID-19; this exceeded the expected percentage (0.9%) (p<0.001) (Figure 2). Among detained persons, 1,214,462 connections were identified, with 3.2% between two persons with COVID-19, which was also significantly higher than the expected 1.5% (p<0.001) (Figure 2). The observed rate decreased to 1.0% when the divisions experiencing the highest clustering related to intentional movement as detained persons with COVID-19 were removed from the network (e.g., to RTU, division 16, or offsite locations). In March, as the number of persons with COVID-19 in the Cook County Jail was increasing, the mean number of interactions between staff members with COVID-19 (377) was significantly higher than that between staff members with negative test results (321) (p<0.001). This difference was not statistically significant in April, when the number of persons with COVID-19 in Cook County Jail was declining.

## Correlation of Positivity and Number of Connections by Division

**FIGURE 3:**
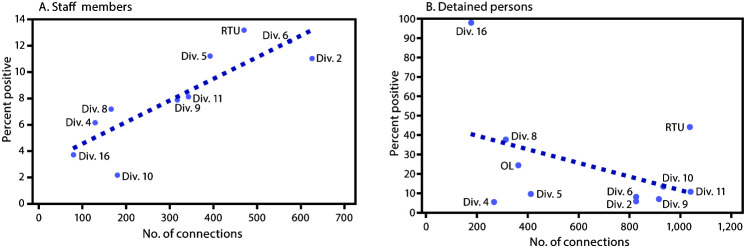
Correlation* between percentage of staff members (A)^†^ and detained persons (B)^§^ with COVID-19^¶^ with number of connections for all divisions — Cook County Jail, Illinois, March 1–April 30, 2020 **Abbreviations:** COVID-19 = coronavirus disease 2019; OL = offsite location; RTU = residential treatment unit. * Staff members: r = 0.86, p< 0.05; detained persons: r = −0.43, p = 0.20. ^†^ A total of 3,278 connections were identified for staff members among all divisions with 198 with COVID-19 cases, as reported to the Cook County Sheriff’s Office. Connections were defined as having at least one shift in a given division during the study period; division connections are not mutually exclusive, so staff members who worked at least one shift in multiple divisions are represented. r = 0.86, p< 0.05 ^§^ A total of 6,056 connections were identified for detained persons among all divisions, with 472 COVID-19 cases. Connections were defined as having at least one bed or cell assignment in a given division during the study period; division connections are not mutually exclusive, so detainees with an assignment in more than one division are represented. ^¶^ COVID-19 cases were defined as detection of SARS-CoV-2 (the virus that causes COVID-19) by real-time reverse transcription–polymerase chain reaction (RT-PCR) testing specimens from in detained persons, and among staff members, as reported symptoms or SARS-CoV-2 positive RT-PCR test results.

Overall, 3,278 connections across all divisions were observed between staff members and divisions, ranging from 80 connections in division 16, to 625 in division 2 (Figure 3). The percentage of staff members with COVID-19 ranged from 2% in division 10 to 13% in RTU. A positive linear relationship was identified between the percentage of staff members with COVID-19 and the number of connections, by division (r = 0.86, p<0.05). Among detained persons, 6,056 unique connections were calculated (Figure 3). The number of unique connections ranged from 266 in division 4 to 1,037 in division 11, with percentage of detained persons with COVID-19 ranging from 6% in division 4 to 44% in RTU, and 98% in division 16.

## Discussion

Network analyses and visualization of a large outbreak of COVID-19 at Cook County Jail demonstrate the complex transmission dynamics that can propagate disease spread, especially in a congregate setting. Connections among persons with COVID-19 occurred more often than expected in staff members and detained persons (p<0.001). The observed connections among detained persons with COVID-19 were significantly lower than expected when considering only the housing units in which initial transmission occurred, suggesting the systematic isolation of those with COVID-19 is effective at limiting subsequent transmission. These findings support the importance of rapid detection and isolation of persons with COVID-19 and limitation of movement between divisions as critical elements in reducing spread (*6*,*7*).

The proportion of connections and number of unique connections by division among staff members with COVID-19 was higher than expected. The correlation between the percentage of staff members with COVID-19 and the number of unique connections by division also demonstrated a strong positive relationship (p<0.05). This likely reflects transmission among staff members; however, additional SARS-CoV-2 exposures not recorded in this analysis might also contribute, including staff members returning to their household and community after each work shift. The higher than expected percentage of staff members with COVID-19 reinforces the need for cohorting staff members (i.e., keeping groups together), maintaining consistency in shift assignment locations, and enforcing mask use for source control.

This is the first known report using network analyses and visualization techniques to describe a COVID-19 outbreak in a U.S. correctional or detention facility. Use of network analyses in China revealed disease occurring in clusters (*8*). Another network study estimated and visualized pandemic risk by calculating worldwide connectedness using the newly confirmed COVID-19 case report counts (*9*). These studies demonstrate the capability of a network approach to supplement traditional investigations and provide timely evidence to inform mitigation strategies and policy decisions.

The findings in this report are subject to at least four limitations. First, the networks described in this report were generated by time- and location-based connections among persons, which might not cover other types of disease transmission, such as fomite spread. Second, for this study, data for staff member work shift dates and locations were only available for CCSO staff members with assignments in housing divisions and not for those in functional roles (e.g., transportation or central kitchen), or other non-CCSO staff members on site (e.g., volunteers, and contractors). Persons not included in the staff member shift data might have interacted with persons in the staff member network. Third, staff members were not systematically tested; thus, this analysis possibly underrepresents the number of staff members with COVID-19 and their connections. In addition, although detained person and staff member networks are displayed separately to more easily visualize the patterns, interactions between these groups likely play an integral role in transmission in a detention facility. Finally, COVID-19 contacts and exposures occurring outside the jail or in the surrounding community, or from staff members with asymptomatic COVID-19, were not assessed in this analysis.

Network analyses and visualizations provide insight into disease spread, illustrating the effectiveness of certain control measures. This study demonstrates the consistent use of cohorting among detained persons and suggests effectiveness of employing this strategy. A network-informed approach can identify likely points of high transmission by demonstrating when transmission is higher than expected, allowing for interventions targeted at these groups or locations (e.g., reducing convening of staff members by closing breakrooms or cessation of contact sports).

**Summary**

**What is already known about this topic?**

Network analyses and visualization can provide information about outbreak transmission dynamics.

**What is added by this report?**

Analysis of detained person and staff member movements during a COVID-19 outbreak at Cook County Jail in Illinois found fewer connections among detained persons with COVID-19 than expected, suggesting that interventions and medical isolation practices were effective at reducing transmission. Higher than expected connections were identified in staff member networks, suggesting occurrence of additional transmission and areas of focus for transmission interruption.

**What are the implications for public health practice?**

A network-informed approach can identify likely points of high transmission, enabling targeted interventions to reduce transmission, such as by reducing convening of staff members, closing breakrooms, and cessation of contact sports.

## Footnotes

* These authors contributed equally.

† https://www.medrxiv.org/content/10.1101/2020.07.12.20148494v1.

§ 45 C.F.R. part 46, 21 C.F.R. part 56; 42 U.S.C. Sect. 241(d); 5 U.S.C. Sect. 552a; 44 U.S.C. Sect. 3501 et seq.
